# Patient-Generated Health Data Integration and Advanced Analytics for Diabetes Management: The AID-GM Platform

**DOI:** 10.3390/s20010128

**Published:** 2019-12-24

**Authors:** Elisa Salvi, Pietro Bosoni, Valentina Tibollo, Lisanne Kruijver, Valeria Calcaterra, Lucia Sacchi, Riccardo Bellazzi, Cristiana Larizza

**Affiliations:** 1 Department of Electrical, Computer and Biomedical Engineering University of Pavia, 27100 Pavia, Italy; elisa.salvi01@universitadipavia.it (E.S.); pietro.bosoni02@universitadipavia.it (P.B.); lucia.sacchi@unipv.it (L.S.); riccardo.bellazzi@unipv.it (R.B.); 2 IRCCS Istituti Clinici Scientifici Maugeri, 27100 Pavia, Italy; valentina.tibollo@icsmaugeri.it; 3 Academic Medical Center, University of Amsterdam, 1105 AZ Amsterdam, The Netherlands; lisanne.kruijver@gmail.com; 4 Pediatric and Adolescent Unit, Department of Internal Medicine, University of Pavia, 27100 Pavia, Italy; valeria.calcaterra@unipv.it; 5 Pediatric Endocrinologic Unit, Department of Maternal and Children’s Health, Fondazione IRCCS Policlinico San Matteo, 27100 Pavia, Italy

**Keywords:** flash glucose monitoring, temporal data analysis, temporal abstraction, patient-generated health data, telemedicine, activity tracker

## Abstract

Diabetes is a high-prevalence disease that leads to an alteration in the patient’s blood glucose (BG) values. Several factors influence the subject’s BG profile over the day, including meals, physical activity, and sleep. Wearable devices are available for monitoring the patient’s BG value around the clock, while activity trackers can be used to record his/her sleep and physical activity. However, few tools are available to jointly analyze the collected data, and only a minority of them provide functionalities for performing advanced and personalized analyses. In this paper, we present AID-GM, a web application that enables the patient to share with his/her diabetologist both the raw BG data collected by a flash glucose monitoring device, and the information collected by activity trackers, including physical activity, heart rate, and sleep. AID-GM provides several data views for summarizing the subject’s metabolic control over time, and for complementing the BG profile with the information given by the activity tracker. AID-GM also allows the identification of complex temporal patterns in the collected heterogeneous data. In this paper, we also present the results of a real-world pilot study aimed to assess the usability of the proposed system. The study involved 30 pediatric patients receiving care at the Fondazione IRCCS Policlinico San Matteo Hospital in Pavia, Italy.

## 1. Introduction

According to the report presented by the World Health Organization (WHO) in 2016, diabetes is a high-prevalence disease, affecting 8.5% of the global population [1]. Diabetes may either prevent the pancreas from producing insulin or prevent the body cells from responding to insulin properly. In both cases, diabetes leads to an alteration in the patient’s blood glucose (BG) level, which needs to be controlled through a combination of diet, physical activity, and medication [1,2].

To optimize therapy for individuals, diabetologists need to monitor their BG values over time. Recently, new devices for continuous glucose monitoring (CGM) have been proposed. This reduces the need to perform BG tests using the traditional glucometer, while providing deeper insight on the patient’s BG trends by monitoring the BG value around the clock [3]. Intuitively, deeper understanding of the patient’s glycemic profile results in a better ability to maintain the BG value in a target range, thus reducing the number or duration of the episodes of hypoglycemia and hyperglycemia experienced by the patient [4,5]. Several wearable devices for CGM are available on the market. Usually, such devices exploit a subcutaneous sensor, which detects BG concentration in the interstitial fluid. While CGM devices return the BG value continuously, other devices apply flash glucose monitoring (FGM), which means they require a scanner to periodically collect the BG values from the sensor. Generally, FGM and CGM systems are complemented with proprietary software, which allows visualizing reports over a pre-defined time interval (e.g., latest 2 weeks), such as the daily glycemic profiles and the ambulatory glucose profile (AGP), an internationally recognized visual representation that combines BG data from multiple days and collates them into a single 24-h period [6]. 

It is well known that the BG profile over the day is influenced by multiple factors related to the patient’s daily routine, which alter the glucose metabolism and/or the body response to insulin [6]. Besides the consumption of meals and the therapy intake, such factors include, for example, physical activity and sleep, in terms of both quality and quantity. In fact, low sleep quality may cause hyperglycemic effects up to several hours after the awakening. Physical activity may lead either to hypoglycemic episodes or to hyperglycemic episodes up to 48 h afterwards [7]. Since their effects on the BG value do not run out immediately, it is fundamental to keep track of the patient’s sleep and activity over time. Nowadays, this is possible thanks to the use of activity trackers that can be comfortably worn by the patient around the clock. Several brands of activity trackers are available, including Fitbit [8], Polar [9], and Garmin [10]. Regardless of the brand, activity trackers usually record multiple parameters related to the user’s sleep, including start time, end time, and number of night-time arousals. They also distinguish deep sleep from light sleep, although the accuracy of this classification is not high [11]. With regard to physical activity, they record start time, end time, and intensity for each detected workout (i.e., activity lasting more than a threshold duration, which may be brand-specific). They also provide a summary of the overall daily activity, including the total number of steps. In addition, most trackers also monitor the subject’s heart rate (HR) continuously. Collecting data from diabetic patients wearing both a BG monitoring device and an activity tracker may help to better understand the relationship between BG values and HR, which is a debated research topic in the literature [12,13,14].

The importance of providing innovative approaches, able to take into account the most recent technological solutions and to promote integrated care by involving both patients and care providers, has been recently identified as one of the main points of action by the European Diabetes Forum [15]. The need to complement the glycemic profile with information on the subject’s lifestyle has been recently discussed by Rodríguez-Rodríguez et al. [7]. In their work, the authors monitored a diabetic patient (male, 25 years old) for 2 weeks, using both the Abbott FreeStyle Libre system and a Fitbit activity tracker, which collected information on the subject’s sleep and physical activity. The subject was also asked to report meals and insulin intake. The graph integrating all the collected data helped interpreting BG values in relation to the patient’s lifestyle factors, which are known to influence the subject’s status.

Since the interest in integrating the BG profile with lifestyle information has been increasing, smart applications to facilitate data integration are needed. For example, the web-applications presented in [16,17] allow the patients to autonomously report performed physical activity, insulin intake, and details on the consumption of carbohydrates. The collected data can be accessed both by the patient and by his/her diabetologist, who can remotely monitor the progress of the patient in diabetes management. Using the web-application developed by Hidalgo et al. [16], the patient can also share with the clinician the results of a set of clinical tests (e.g., eye examination) that are usually carried out to identify the onset of possible complications due to diabetes. However, rather than continuous BG monitoring, these systems consider data collected using a glucometer and a manual data entry is required for the other parameters. Such manual daily data collection can fatigue the patients, who, in the long run, might stop providing this data [18,19]. Thus, since the use of wearable sensors has intensified in recent years, the most recent applications designed for chronic patients aim to automatically collect as much data as possible from such devices. For example, several commercial platforms focus on supporting patients affected by Type 2 diabetes [20,21,22,23,24] by tracking both BG measurements collected using glucometers and activity data collected by trackers. Such applications usually provide dashboards for the integrated visualization of the collected information, and sometimes allow the patient to share the data with the physician. Recent applications intended for Type 1 diabetes collect and visualize CGM or FGM monitoring data [3]. Such applications usually provide daily reports, including the number and the duration of hypoglycemic and hyperglycemic episodes, possibly filtered by time of occurrence (e.g., nighttime or daytime). They also provide other summaries, such as AGPs. However, few applications integrate BG monitoring (either CGM or FGM) with activity and sleep tracking. One of the first solutions proposed in this area was Nightscout [25], an open source project developed by volunteers in 2014 to help the patient, or his/her family, set up a custom system for collecting and visualizing both BG monitoring data and activity tracking. However, this solution was not suitable to all patients, since it required the ability to build and maintain such a system, which includes a website and a database. Other solutions [26,27] include commercial (integrated) systems, which include a sensor for CGM and proprietary software to process the collected data. Such systems gather information on physical activity using proprietary insulin pumps [27], or commercial activity trackers [26]. To our knowledge, such systems provide an integrated visualization of data, but do not provide any tool for performing advanced analyses that combine data from both the sources. The same consideration holds for recent applications, such as the M:Diabetes mobile application [28], which was not developed by the same companies that produce the sensors, and aims to be compatible with multiple brands of sensors, insulin pumps, and wearable devices for activity tracking. 

Recently, we have developed AID-GM (Advanced Intelligent Distant—Glucose Monitoring), a web application that allows diabetic patients and their clinicians to share and analyze FGM data [29,30]. In this work, we describe the extended version of AID-GM, providing advanced data analyses that combine FGM data with the information collected by Fitbit activity trackers (i.e., HR profile, activity, and sleep tracking). We also evaluated the usability of our application in a real-world pilot study involving 30 pediatric patients receiving care at the Fondazione IRCCS Policlinico San Matteo Hospital in Pavia, Italy.

## 2. Materials and Methods

**Figure 1:**
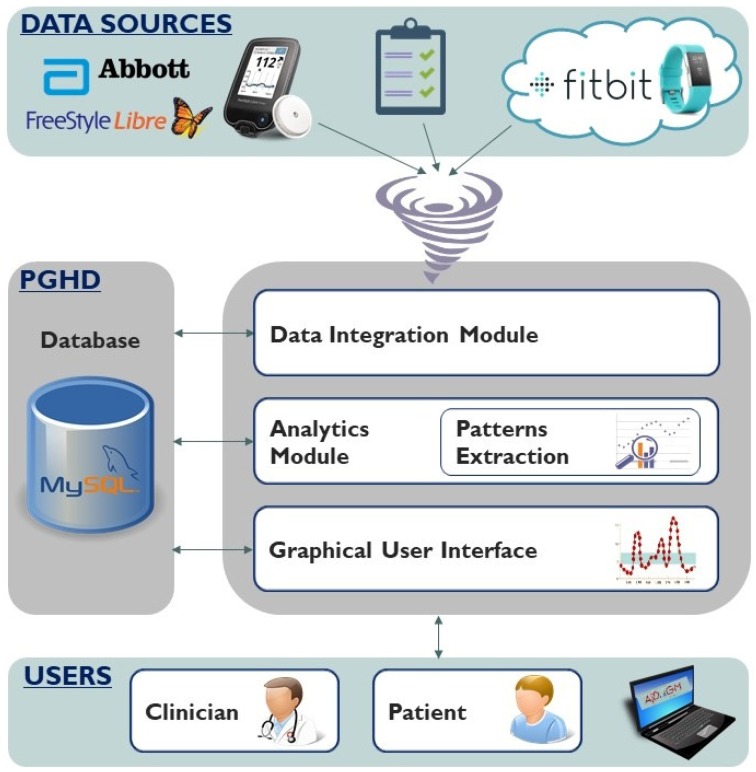
The AID-GM architecture.

AID-GM is a web application mainly developed in Java (AID-GM is a web application mainly developed in Java, and integrated with JavaServer Faces, Hibernate, and MySQL technologies. We chose to develop the system as a web application to make it accessible from any device with an internet connection and a browser, regardless of its type and operating system. We integrated the JavaServer Faces framework since it facilitates the development of the components in the user interface, while we used MySQL as the database manager system since it is designed for web applications, it is open source, and it offers detailed documentation for configuring the service. We integrated the Hibernate framework since it facilitates converting the java objects manipulated by the AID-GM system into the tabular contents stored in database, and vice versa.). The system architecture, shown in Figure 1, includes a data repository integrating the heterogeneous data collected from different sources, which we call PGHD (Patient-Generated Health Data), and three main components, which will be described in the following paragraphs.

### 2.1. Data Integration Module

AID-GM manages several kinds of PGHD. The patient’s glycemic profile can be collected using FGM or CGM systems. Although AID-GM is designed to be independent of the specific glucose monitoring device, in this work we show an application on the Abbott FreeStyle Libre FGM system [31]. This system has been approved for use in pediatric patients, who represent the target of our study [32]. Like all FGM devices, the FreeStyle Libre system includes a reader, that must be used to scan a sensor positioned on the upper arm of the patient. Such sensor must be scanned at least every 8 h and provides one BG measure every 15 min. The reader can also be used to store details on insulin intake, meals, and physical activity. The system includes a proprietary application, recently made available also as a web portal (https://www2.libreview.com/), to download data from the scanner, visualize it, export it to a text file, and produce a PDF summary report. To collect information on the subject’s HR, daily activity and sleep, AID-GM uses a Fitbit activity tracker. The tracker is connected to a mobile application that, upon synchronization, transfers the collected data to the Fitbit cloud. Any application can retrieve data from the cloud through HTTP requests, provided that it has been authorized by the data owner through the Oauth2 Protocol (https://dev.fitbit.com/build/reference/web-api/oauth2/). In AID-GM, data coming from Fitbit are automatically retrieved from the cloud every night. A daily summary of the measured activity, reporting the total amount of time in which the patient has been moving, is stored. For each workout, we store start time, intensity, and duration. For each sleep record, we memorize when a subject falls asleep, the time he/she wakes-up, the amount of time in which the subject was awaken in bed, and the amount of time in which the subject’s sleep was restless. The subject’s HR profile, which includes one HR measurement per minute, is also stored. Finally, information on the subject’s habits, including the meal schedule on each day of the week, is collected directly through the AID-GM system interface.

**Figure 2:**
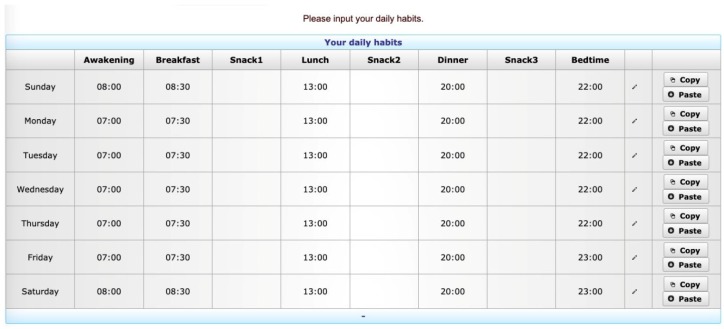
Form to provide, for each day of the week, patient’s usual time schedule concerning daily habits.

The Data Integration Module is the component responsible for acquiring and pre-processing the described data. Data acquisition requires the patient’s collaboration, with a level of commitment that depends on the type of data. Patients’ habits are collected at registration and can be modified if the patient thinks it is necessary. After registering, the patient is asked to fill in a form to provide, for each day of the week, his/her usual time schedule concerning primary meals, snacks (i.e., snack after breakfast, after lunch, and after dinner), and sleep (Figure 2). BG data from the FreeStyle Libre system should be periodically uploaded into AID-GM by the patient, using a dedicated form that takes as input the text file produced by the Abbott software. Each row in this file describes one event, which can be a BG measurement (automatically measured or manually scanned by the patient using the reader), a bolus of insulin, a health-related issue, or a meal. Each event is defined by its time of occurrence and by other attributes that depend on the event type. For BG events, the measured BG value is specified, while for insulin events the bolus dosage, inserted by the patient, is reported. A health-related event includes a textual description of the issue, while the meal event has no additional attributes. The Data Integration Module stores the events in a MySQL database (DB).

**Figure 3:**
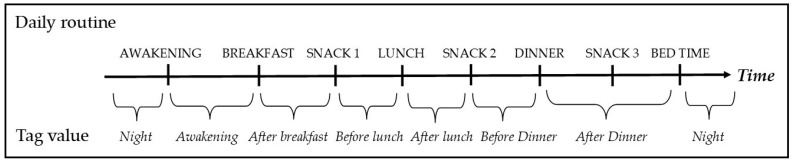
Assigning the profile tag to BG events. The tag value (bottom) is assigned by comparing the time of occurrence of the considered event to the usual time of the patient’s daily activities (top).

One of the most important tasks of the Data Integration Module is tagging each BG and HR measurement to contextualize them within the day of the subject. This can be done both by considering the profile information provided by the patient and, for patients wearing an activity tracker, by using information about workout and sleep. We will refer to tags defined using patient profile as *profile tags*, and to tags defined using Fitbit data as *Fitbit tags*. Figure 3 shows the process of computing the *profile tag*. 

The set of possible values for the *profile tag* includes *awakening*, *after breakfast*, *before lunch*, *after lunch*, *before dinner*, *after dinner*, and *night.* The *night* tag value is assigned when the time of occurrence is between the usual bedtime and awakening time.

The *Fitbit tag* can assume the following values: *sleep*, *workout*, *routine*, and *NA*. The *sleep* and *workout* values are assigned when an event (BG or HR) occurs during a tracked sleep session or during a tracked workout, respectively. The *routine* value is assigned when the event occurs in an instant in which the patient is not sleeping and is not training. The *NA* value is assigned to each BG event occurring when the patient is not wearing the Fitbit tracker. In particular, we assume that the subject was not wearing the tracker at time *t_i_* if no HR measurements are available in the interval [*t_i_* − 5 min; *t_i_* + 5 min].

For each event, both the *profile tag* and the *Fitbit tag* are stored into the database. When the BG data is analyzed, only one tag is considered to contextualize the measurements during the day. When patients do not wear the Fitbit tracker, the Fitbit tag value is NA, and the *profile tag* is used. When both tags are available, the user can decide which of the two tags has to be considered. In this case, the *Fitbit tag* may be preferable, since it provides more accurate contextualization of the events.

### 2.2. Analytics Module

As described in our previous work [29], AID-GM is integrated with the Java Time Series Abstractor (JTSA) library [33], a framework recently developed at the Department of Electrical, Computer and Biomedical Engineering of the University of Pavia, Italy, by some of the authors of this paper. JTSA extracts qualitative patterns from time series of measurements using Temporal Abstractions (TA) [34,35,36,37,38]. In agreement with diabetologists, in [23] we defined a set of 6 knowledge-based patterns that are relevant for evaluating the diabetes outcome (i.e., Hypoglycemia, Hyperglycemia, BG Increasing, BG Decreasing, Rebound Effect, and Down Effect, as described in Table 1). While the patterns described in our previous work aimed to detect trends of interest in the subject’s glycemic profile, in this work we focus on integrating the patient’s lifestyle into the analysis, by exploiting the data collected by the Fitbit tracker. This was achieved by extending the set of implemented patterns and by providing new visualization options, that complement the patient’s glycemic profile with information on sleep and physical activity. In addition, in the new version of the AID-GM system it is possible to contextualize the search for glycemic patterns within the patient’s day, thanks to the *Fitbit tag*. For example, it is possible to search for the occurrence of hypoglycemic events specifically during the subject’s sleep or during the workout. 

The set of patterns was extended with 2 univariate patterns on HR (i.e., bradycardia and tachycardia) and 2 multivariate patterns that combine HR and BG trends. Table 1 lists all the patterns currently available in the AID-GM system. We distinguish between basic patterns, extracted from one single time series, and complex patterns, which consist of a combination of patterns, potentially extracted from different time series. For each pattern, the table reports the input data and a graphical representation of the behavior of interest. For Hypoglycemia, Hyperglycemia, Bradycardia and Tachycardia, the threshold value is patient-specific and personalized by the clinician using the system graphical user interface.

The algorithms for pattern detection were tested and validated on simulated data sets in our previous works [33,39,40].

### 2.3. Graphical User Interface

**Figure 4:**
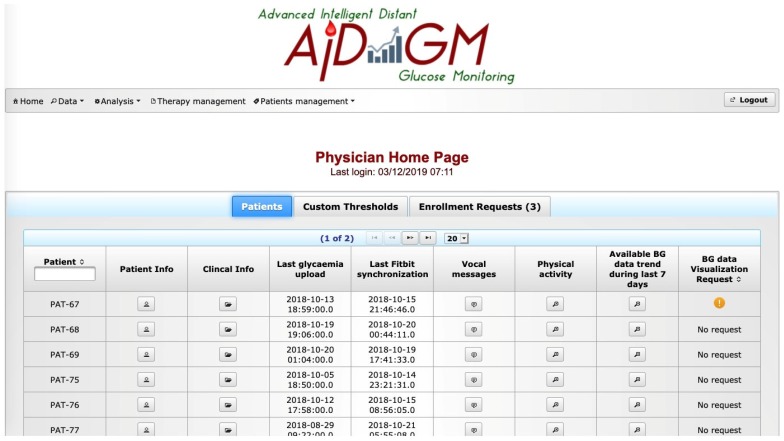
Physician’s home page.

The AID-GM graphical user interface (GUI) allows the patient and the physician to take advantage of the functionalities provided by the two modules described above. Figure 4 shows the physician’s home page, on which a summary of the patients’ activities (last BG data upload, last tracker synchronization, last week BG summary, etc.) and relevant information are provided. 

Table 2 lists all the available system functionalities, specifying to which user/s they are addressed. The *status* column identifies new and upgraded features with respect to the previous version [29]. 

**Figure 5:**
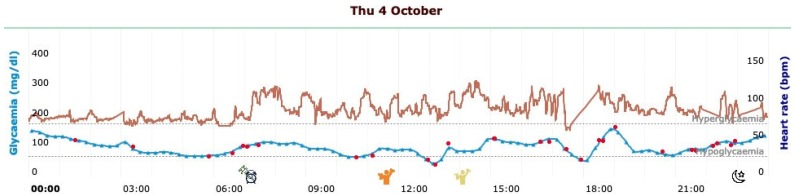
Daily profile, complemented by information on the subject’s sleep and workout.

As shown in Table 2, one of the most interesting new features is the possibility of accessing several reports that integrate BG and activity tracker raw data, summarizing both the BG levels and the patient’s lifestyle. It is also possible to get a synthetic overview of the metabolic control through the visualization of the temporal patterns described in Table 1. All these data views are meant to support patients and physicians in taking decisions about timely changes of lifestyle, diet, or therapy and, more generally, in a more informed disease management. In particular, the system provides four main types of visualizations: *Daily profile*, *Lifestyle summary*, *Physical activity summary*, and *Pattern visualization*. First, AID-GM provides a *Daily profile*, combining BG and HR daily profiles with additional events like sleep start, sleep end, workout, insulin dosage, and meals (Figure 5). 

**Figure 6:**
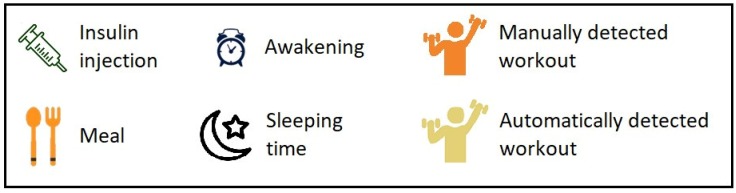
Legend of the additional events related to the patient’s lifestyle.

In Figure 5, the BG profile is represented in blue, and the HR profile is represented in red. On the BG chart, the red dots represent the BG values shown to the patient by the FreeStyle Libre reader when used to scan the sensor. On the timeline, colored icons represent the previously described additional events. The legend of the supported additional events is shown in Figure 6.

**Figure 7:**
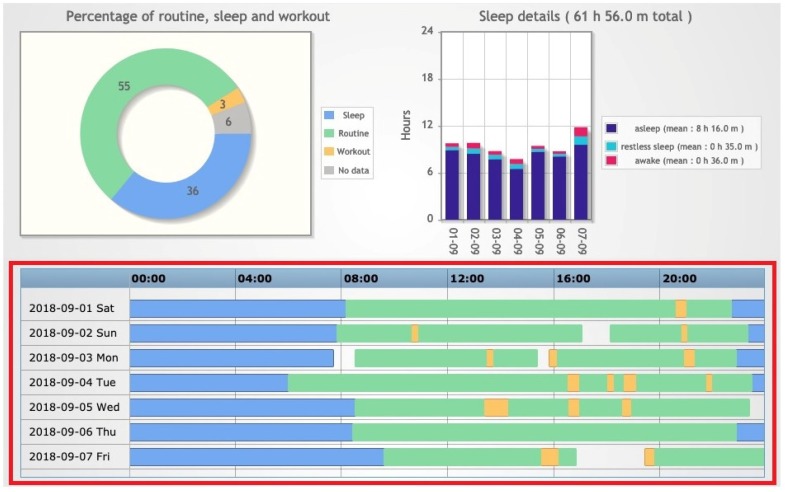
Example of a subject’s Lifestyle summary recorded during holidays.

**Figure 8:**
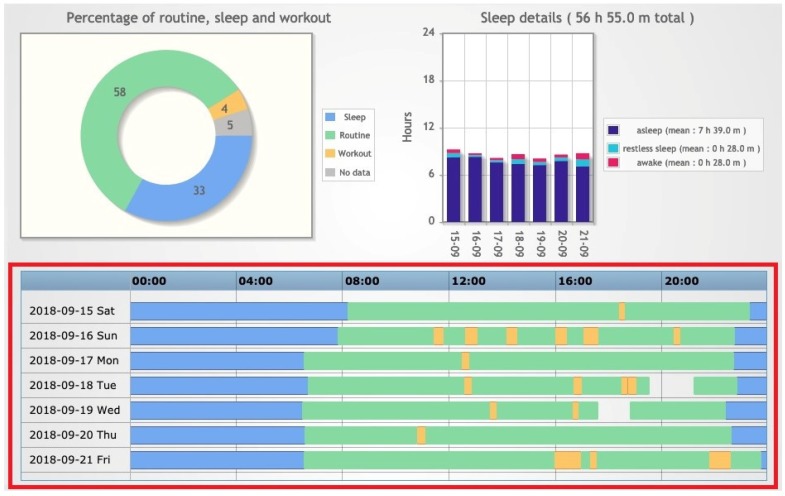
Example of a subject’s Lifestyle summary recorded during the school period.

The application also provides a *Lifestyle summary*, consisting of several charts that give an overview of the activities and lifestyle of the patient over a selected period (Figure 7 and Figure 8). The time frame of the summary can cover one day, one week, one month, or a user-defined period. A section of particular interest is the patient timeline (highlighted in red), where the different activities registered by the tracker related to a specific day are shown on a single timeline with different colors. The visualization of the timelines related to a specific period can easily point out if the patient lifestyle was regular in terms of sleep and physical activity. For example, Figure 7 and Figure 8 report the timelines of a patient in two different weeks, the first one during holidays and the second one during the school period. The timelines in Figure 7 show irregular activities and sleep habits coherent with the vacation context. During the school period, the patient shows more regular habits during school days than in the weekend (Figure 8).

**Figure 9:**
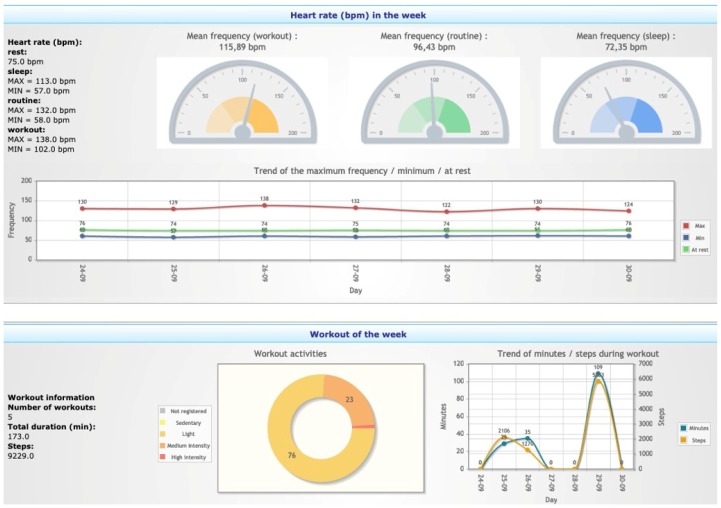
Physical activity summary visualization with the subject’s HR profile and workouts.

The *Physical activity summary* visualization (Figure 9) shows an overview of the HR measurements (top) and the workouts performed by the subject (bottom) in a selected period.

**Figure 10:**
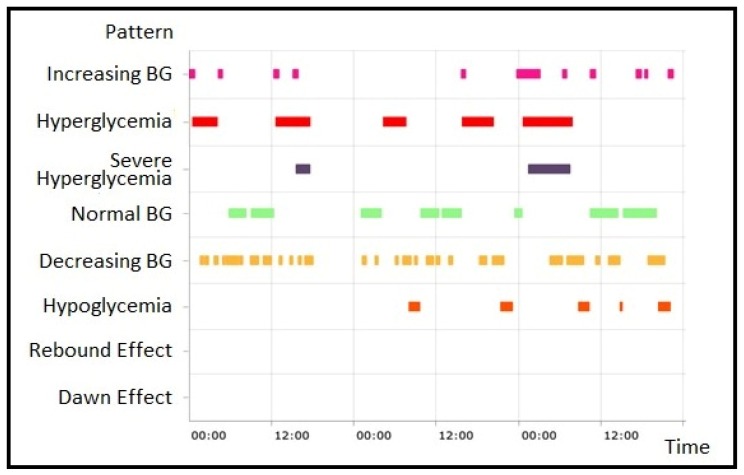
Pattern visualization for the single patient.

**Figure 11:**
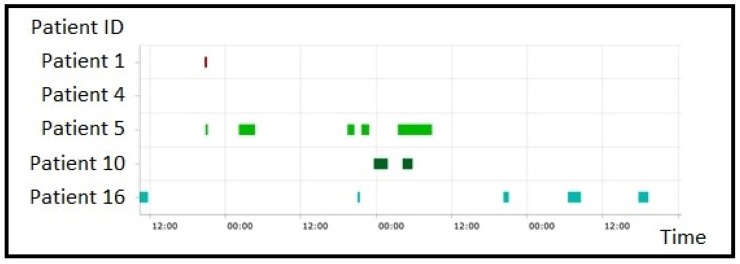
Pattern visualization for a group of patients.

**Figure 12:**
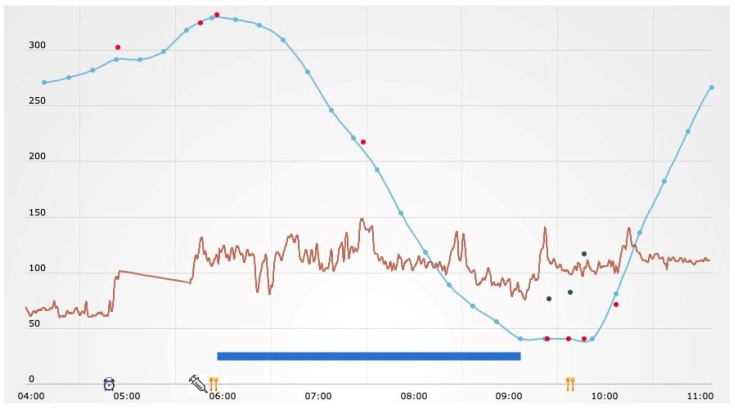
BG and HR profiles related to a selected pattern occurrence. On the timeline, the blue line represents the time interval in which the selected pattern (in this case, decreasing BG value) occurred.

The last type of visualization is the *Pattern visualization*. The GUI helps the user through the process of selecting patients, patterns and the period of interest for the analyses, and then visualizing the obtained results, i.e., the time intervals at which the selected patterns occurred, presented in the form of colored bars. Two examples of this visualization are shown in Figure 10 and Figure 11. In Figure 10, all the patterns of a single patient are displayed, while in Figure 11 a single type of pattern for a group of patients is shown. For each pattern occurrence, the corresponding colored bar links to the *Daily profiles* charts related to the time interval of that pattern occurrence (Figure 12), supplemented by information on the subject’s activities in that time interval (e.g., sleep, workout, and insulin intake). This combined visualization can help the physician to quickly evaluate and identify the problems in the metabolic control of a patient, or a group of patients.

## 3. Results

The usability of the system was evaluated in a 6-month pilot study carried out in collaboration with the Pediatric Diabetology outpatient service of the IRCCS Policlinico San Matteo hospital in Pavia, Italy. The study was approved by the Institutional Review Board (IRB) of the hospital. The subjects enrolled in the study were already being followed by the center and were already using the FreeStyle Libre device for monitoring their BG. The patients involved in the study were asked to use the system to periodically upload their BG data and, if they wanted to, for visualizing information related to BG profiles. Some of the patients were wearing a Fitbit tracker, and provided their consent to download their Fitbit data and analyze it together with their BG data. After 2 and 6 months from enrollment, patients were asked to fill in the System Usability Scale (SUS), a well-known 10-item questionnaire that asks users to assess the perceived ease of use of the system and their willingness to continue using it in the future [41]. For patients aged less than 18 years, the questionnaires were filled in by their parents, whereas patients older than 18 filled in the questionnaires themselves. Patients were then treated following the usual practice. In addition to patients, 3 doctors were also asked to fill-in the SUS questionnaire at the end of the study. Of the 30 patients originally enrolled in the study, 3 dropped out at the beginning of the study, leaving us with a sample of 27 subjects, whose characteristics are shown in Table 3. As shown by the statistics on the duration, not all the patients were continuously using the monitoring devices.

To further characterize the patients’ population, we used AID-GM to perform an analysis of the BG profiles through pattern detection. Table 4 provides a snapshot of the total number of BG patterns detected in the dataset using the system. The patterns were computed on all the patients taking part in the study. As already stated, thresholds for defining hypoglycemic and hyperglycemic episodes are patient-specific and are defined by the diabetologists through the system. *Severe Hypoglycemia* is defined as an episode of at least one measurement of BG < 50 mg/dL, whereas *Severe Hyperglycemia* is defined as an episode of BG > 250 mg/dL. *BG Increasing* and *BG Decreasing* episodes are defined as a variation of the BG level of at least 15 mg/dL every 15 min, lasting for at least 35 min. The values of the parameters needed to compute the patterns were agreed with the physicians and validated on a sample of the available time series. Table 4 shows that there are some patterns that happen more frequently than others in our population, and that different patterns have different durations. In particular, *Increasing* and *Decreasing BG* trends are the most frequent of the considered patterns, but the episodes that last longer are the *Hyperglycemia* ones.

**Figure 13:**
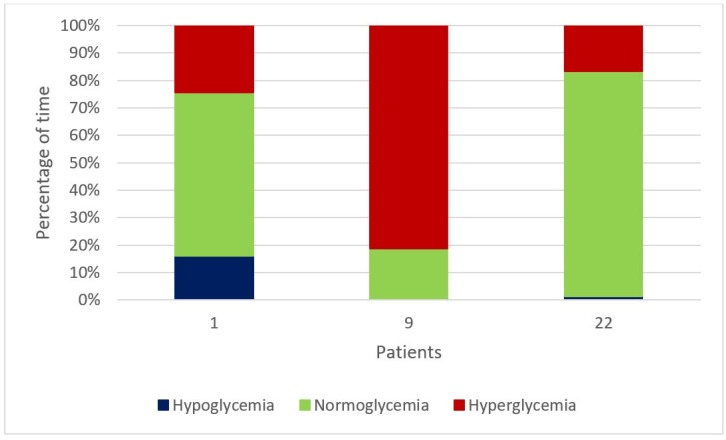
The computation of percentages of time spent in Normal BG range, Hyperglycemia, and Hypoglycemia can easily identify different types of patients.

The patterns on BG can also be used as indicators of glycemic control in our patients. As an example, Figure 13 shows the percentage of time spent in Normal BG range, *Hyperglycemia*, and Hypoglycemia for 3 representative patients in the study. From this picture it is possible to easily identify a subject who spends the majority of time with high BG values and less than 1% of time in hypoglycemic episodes (Patient 9), a subject who has long periods of normal BG values (Patient 22), and a patient who experiences several hypoglycemic episodes (Patient 1). This type of analysis could allow diabetologists to quickly divide the patients in groups, which can be managed with different interventions or treatment strategies.

To be able to fully exploit the potential of the TA framework, it is interesting to analyze data for the six patients who were wearing both the FGM and the Fitbit devices simultaneously (Patients 1, 2, 5, 6, and 10). In this case, it is possible to extract several additional patterns, involving multiple variables (BG, HR, and tracked sleep).

As an example of a pattern involving BG and sleep, it is possible to compare the patients in terms of episodes of nighttime hypoglycemia. This is very important, especially for children, since it is crucial to quickly identify who are experiencing this pattern more frequently than others. Table 5 presents the number of nights with at least one hypoglycemic episode and the total number of nights, for the six patients. Thanks to the advanced analysis functionalities offered by AID-GM, it was easy for the physician to identify the patients who were more prone to a specific type of episode. For example, comparing Patient 5 to Patient 10, it is possible to see that the first one has used the Fitbit for less nights than the second (56 vs. 165), but the proportion of nights with at least one hypoglycemic episode is higher.

Another interesting analysis, which makes it possible to understand the importance and usefulness of integrating different devices, is the comparison between the patterns extracted by the system when using the *profile tag* or the *Fitbit tag*. Table 6 shows this comparison for the six patients wearing a Fitbit tracker. The Table reveals that, for patients with irregular sleep habits, such as Patient 1, the difference between the number of hypoglycemic episodes can be significant. For critical episodes, such as those of low BG levels during sleep, a proper monitoring is of crucial importance. Moreover, excluding Patient 1, for the other patients the algorithm is not able to detect Dawn Effect without the sleep information detected by Fitbit. 

To evaluate the system use, we analyzed the log files of AID-GM. These files include information about the type of action performed by the user, together with the date and the time of execution. The available types of actions are *Login*, *Logout*, *Data visualization*, *Modify*, *Upload BG data*, and *Find pattern*.

**Figure 14:**
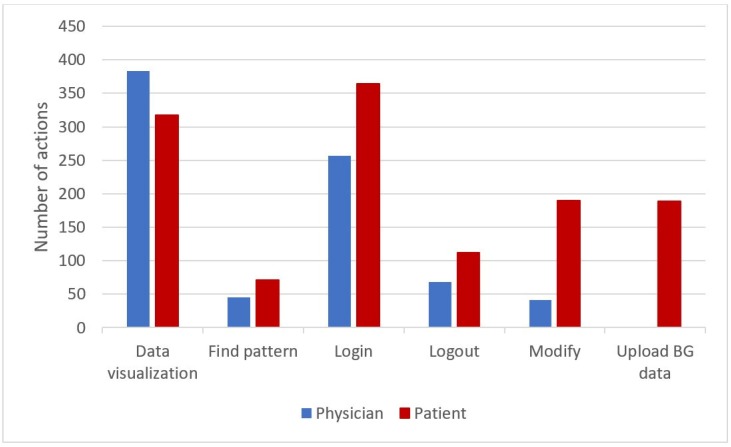
Frequency of actions performed by the AID-GM users in the pilot study.

Figure 14 shows the frequency of each action, distinguishing patients and physicians as user types. As shown by the bar chart, besides the *Login*, the most frequent action performed by both users is the *Data visualization* action. This action refers to the several types of visualization available in AID-GM, which include the visualization of BG profiles and summaries, and the visualization of patients’ demographic and clinical information. As regards *Find pattern*, each occurrence of this type of action may concern the search for single or multiple patterns over a single patient or a group of patients. 

**Figure 15:**
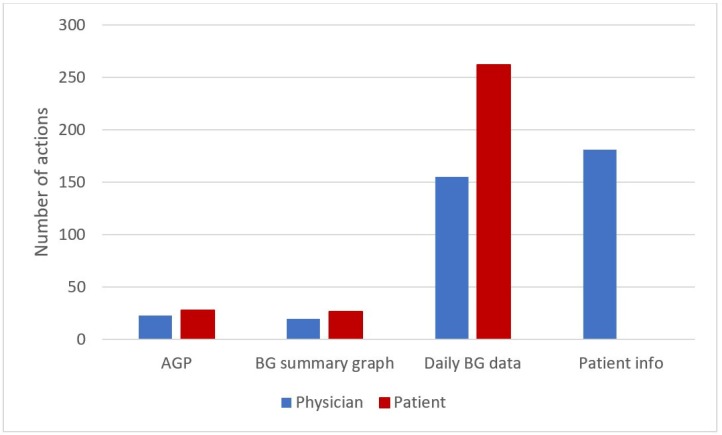
Distribution of the visualization action.

Figure 15 illustrates the detailed distribution of the available visualization actions, indicating that patients are mostly interested in checking their daily BG trends. For doctors, we registered several visualizations of the patients’ information (*Patient info*), and visualizations of daily BG trends.

In addition to the overall number of actions, we also considered the weekly trend, computed as the total number of actions for each week of enrollment. This analysis, reported in Table 7, showed that the average number of actions in the first week was higher than the average number of actions in all the following weeks, both for patients and for physicians. Doctors use the system more during weekdays and in the morning, whereas patients have a more uniform distribution of usage throughout the week and during the day, with an almost equal number of actions in the morning, afternoon, and in the evening.

Table 8 shows the average session and training durations. The duration of sessions was computed as the time interval between a login and a logout when the latter was available, and as the time interval between a login and the last action before the next login, when logout was not available. In fact, when the user does not use the application for more than 30 consecutive minutes, the work session expires automatically, and no logout activity is recorded in the AID-GM system log. Furthermore, since the first access to the system is performed by the patient together with the physician, who trains the new user by illustrating the functionalities of the system, we finally evaluated the duration of the first session for each patient to have an estimate of the training time. 

As anticipated, the system usability was assessed using the SUS questionnaire, which was delivered both to physicians and patients. For patients, we carried out an assessment at 2 months from the beginning of the study and one at the end of the study. The average SUS score at 2 months was 82.6, whereas at 6 months, we registered a slight decrease in the average score, which was 76.4. Even though both scores are considered above average with respect to the threshold of 68 [41], we investigated the obtained results to better explain the reasons for this decrease. First of all, from the analysis of the system logs, we observed that, even though all the patients filled in the questionnaire at the end of the study, 8 of them never used the system after two months. Considering only the patients who performed at least one access after two months of usage, the average SUS score at 6 months was 81.3. For 11 patients belonging to this group, the SUS score increased or remained stable, whereas for 8 patients we registered a decrease in the score. Analyzing the individual questions, the one that we found most critical was the following: “I found the various functions in this system were well integrated”. For this question, 7 patients gave a lower score after 6 months than at 2 months. Perhaps this question was not entirely understood by the patients, because of its technical formulation. Three physicians completed the questionnaire at the end of our study. In this case, we had the maximum SUS scores for all three (100). The motivation for such high scores could partially be due to the fact that 2 out of 3 users also participated in the development of the system. The third user instead started to use the system at the beginning of the study, without any previous knowledge.

## 4. Discussion

In this paper, we presented AID-GM, a web application for managing patients with diabetes that enables the integration of BG and activity data, provides advanced temporal data analysis functionalities, and encourages communication between patients and their care providers. 

Thanks to the Data Integration Module, AID-GM is able to jointly analyze data coming from different sources. This offers several advantages and novelties. First, Fitbit data are used to complement BG measurements providing information about the actual lifestyle of a patient in terms of sleep and physical activity. As shown in Figure 5 and Figure 6, the availability of such information also allows the identification of irregularities in the habits, which could be an important factor not usually included in applications for monitoring diabetic patients, for interpreting the metabolic response. In addition, the availability of HR data offers the possibility to perform multivariate analysis to investigate the relation between the reaction of HR to variations in BG levels. To our knowledge, AID-GM is the first application that uses Fitbit data to contextualize BG data and carry out advanced multivariate temporal analysis. To fully appreciate the novelty of the AID-GM application, it should be compared with the other tools available for collecting and analyzing CGM or FGM data, rather than tools that consider data collected using glucometers. As anticipated in the introduction, few applications are available for analyzing CGM or FGM data, and fewer applications integrate it with information on the subject’s sleep and activities collected automatically from wearable devices. Among these applications, some solutions [25] are not ready to use, while others [27] are not applicable to all patients, since they gather activity data from proprietary insulin pumps. We believe that collecting data from widely used activity trackers, instead of specific insulin pumps, may make the solution accessible to a higher number of patients. Several efforts are converging towards this goal. For example, we are aware that Fitbit and Medtronic reached an agreement, that resulted in a mobile application for visualizing both Fitbit data and BG data collected by a professional device for CGM monitoring, iPro™2 [42,43]. However, the iPro™2 device is only intended for diagnostic purposes, and not for long-term remote monitoring. We are aware of another application that is aiming to integrate FGM data with Fitbit data [28]. However, this feature is not yet listed among the functionalities available in the current version of the system. To the best of our knowledge, there are no available long-term solutions that offer multiple data views combining both BG and activity data, and that allow both the patient and the clinician to perform multivariate analysis through pattern detection. Thus, we believe that the integrated view offered by AID-GM opens a new perspective on the exploitation of PGHD, which can be used to perform, in a realistic day-by-day setting, analyses that have traditionally been limited to clinical studies. In particular, for adolescents and for parents of children, AID-GM can guide clinical decisions regarding therapy and could represent an interactive system to learn diabetes self-monitoring potentially enhancing patient care, especially immediately after the disease onset. Moreover, the automatic collection of BG and lifestyle data ensures an objective picture of the real patient’s behavior and metabolic response, preventing from errors that can occur when manually reporting the daily diary both in paper and electronic form.

AID-GM includes an Analytics Module designed to carry out advanced data analytics through Temporal Abstractions. Thanks to this technique, it is possible to formalize the qualitative patterns that clinicians frequently already have in mind, and automatically extract the behaviors of interest from the available data. The patterns that can be extracted range from basic and univariate to complex and multivariate, like the Dawn Effect. Despite its clinical relevance, the Dawn Effect is difficult to manually detect in a large amount of data without accurate information on the sleep and wake up time. Thanks to AID-GM, it is possible to formally define the pattern, and automatically extract it from the available data.

In addition to the flexibility offered by JTSA for patterns definition, another convenient feature of AID-GM is related to the temporal filtering functionalities available for data exploration and analysis. In fact, differently from other available tools that provide a view on data over fixed time ranges, the patterns can be dynamically extracted on user defined time-windows or on specific days of the week, which can be selected over the complete follow-up of the patient. Moreover, for each detected pattern, it is possible to inspect the raw data it was generated from (Figure 10), thus offering complete control over the obtained results. These features enable the users to dynamically perform their analyses, potentially exploiting the tool to discuss the progression of the disease together during encounters or comparing the metabolic control in different periods and day or week slices (working days/weekend, sleep, routine, workout).

Finally, differently from other tools, AID-GM enables the extraction of temporal patterns over a group of patients. This can be useful in two ways. First of all, considering a single pattern, the tool can be used to highlight those patients who experience it more frequently. Second, considering multiple patterns, this feature allows the detection of the groups that need closer attention. In this paper, we have shown several examples of how the analysis results can be used to identify groups of critical patients. In the future, a careful analysis of the occurrence of the patterns over a larger cohort of patients could be used to propose some pattern-based indicators to quickly characterize patients based on their metabolic control.

As shown by the results of the SUS questionnaires, the AID-GM system was considered to be user-friendly both by patients and physicians during the real-world pilot study. This result is particularly positive, considering that the tool introduces a change in the workflow related to the FreeStyle Libre data management for both user types. Before the introduction of the system, patients used to download their BG summary report from the FreeStyle system only before periodic face-to-face encounters, in order to discuss their BG profile with the diabetologist. During the visit, the diabetologist would consider those reports in order to plan future interventions and treatment. Even though the FreeStyle software offers the possibility of downloading the time series of the collected BG measures from the patient’s sensor, this was rarely performed during encounters because of the lack of tools for data analysis. During the pilot study, the possibility of remotely sharing BG data with the clinician motivated the patients to download their data frequently, and to upload it into the AID-GM system on a regular basis. The obtained SUS scores and the analysis of the actions performed by the users point out that patients believe it is worth investing time in uploading data to the system to facilitate remote monitoring of their health condition, and possibly to receive better care. The advantage derived from the use of the AID-GM system is even more evident when considering health care personnel, who are able to gain deeper insights into how the patient’s condition has evolved between visits and, potentially, during the entire follow-up of the patient. While the data stored within the FreeStyle Libre device include only the most recent three months, the AID-GM system maintains the complete history of BG data uploaded by the patients, of the Fitbit data, and any other information that was provided through the FreeStyle system’s BG reader, like insulin intake and meals. This allows the collection of long histories, which may be used in the future to train and personalize data-driven systems for supporting decision making in the context of Type 1 diabetes.

The work presented in this paper describes ongoing research, which still has some limitations that will be addressed in future work. First of all, AID-GM currently works on a set of temporal patterns that is predefined on the basis of clinical knowledge. Even though this set might be arbitrarily and easily extended, the inclusion of a new pattern would need to be hypothesis-driven. Using only this approach prevents the system from discovering potentially unknown behaviors. Given the peculiarities of the data being considered, which have only recently started to be collected and jointly analyzed, the possibility of mining unknown multivariate patterns is a desirable feature. For example, it is widely acknowledged that lifestyle habits have an impact on metabolism, but evidence on how this reflects subjective parameters such as HR, BG, and sleep is still lacking. Considering our previous work on temporal association rules mining [39], we are planning to include a module with this functionality in the AID-GM system.

Currently, the Data Integration Module retrieves data from the sensors by using the Application Programming Interfaces (APIs) made available by Fitbit, and dedicated readers specifically designed for storing the data in the AID-GM DB. Even though this custom solution fits well with the system deployed for the pilot study, we are aware of the importance of devices interoperability, especially in remote patient monitoring scenarios. Such importance has been acknowledged by several international projects, such as the open mHealth to FHIR project promoted by HL7 [44], or the Remote Patient Monitoring supplement to the Technical Framework developed by the IHE Patient Care Coordination domain [45]. Taking into account these initiatives, future work will be devoted to the development of a standardization component within the Data Integration Module, responsible for making the exchanged data available in a standardized format to promote interoperability among devices and other systems.

An additional aspect to be considered is that, in order to have a complete picture of a patient’s condition, it is important to also consider those events, usually reported in paper diaries, which cannot be automatically monitored. Up until now, AID-GM has supported the inclusion of some events (meals, insulin intake, health related issues) that could be recorded manually by the patient using the FreeStyle Libre reader. Our experience, though, is that patients do not frequently enter data through the reader, probably due to the time it takes to access the functionality and store the information. To overcome this limitation and motivate the patients to provide the necessary information, we are working on a mobile app where the patient could record a voice message reporting any relevant event. The system will then automatically process and store the information, integrating it with the rest of the data available for that subject.

Finally, in this study we focused on the assessment of the system from a usability perspective, without analyzing the impact of the introduction of the system from a clinical viewpoint. To this end, a larger study, possibly including both children and adult patients, should be planned. Depending on the study duration, it will be possible to evaluate outcomes such as the time in range, the time in target [46,47,48], or the variations of glycated hemoglobin.

## 5. Conclusions

In this work we developed and tested AID-GM, an application that allows better interpretation of the glycemic profile of an individual suffering from diabetes by analyzing the BG monitoring data contextualized according to the patient’s activities, monitored by a fitness tracker. Although the interest in integrating the BG profile with activity data is shared by the scientific community, to the best of our knowledge, to date, no solution has enabled advanced analyses of time series of BG data taking into account the patient’s daily context.

AID-GM proved to be user-friendly in a 6-month pilot study, in which a group of patients, who were already using both the FGM device and the Fitbit tracker, used our system for sharing monitoring data with their diabetologists. Thanks to its multiple data views, we believe that AID-GM may be a useful support for diabetologists in understanding the patient’s glycemic profile and, consequently, in personalizing the care process for the individual. Moreover, as anticipated in the discussion, the possibility of analyzing a group of patients simultaneously may facilitate the identification of subjects in need for closer attention, favoring prompt intervention.

## Figures and Tables

**Table 1:** Patterns of interest to evaluate the diabetes outcome. Red dots represent BG measurements; blue dots represent HR measurements.

	Pattern	Input Data			Graphical Representation
		BG	HR	Sleep	
**Basic**	Hypoglycemia	•			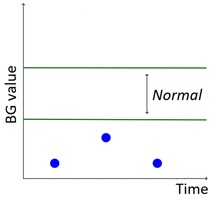
	Hyperglycemia	•			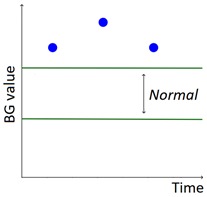
	BG Increasing	•			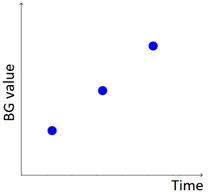
	BG Decreasing	•			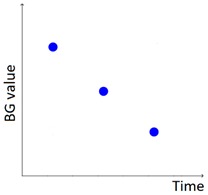
	Bradycardia		•		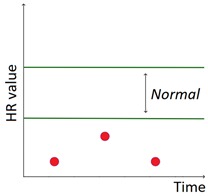
	Tachycardia		•		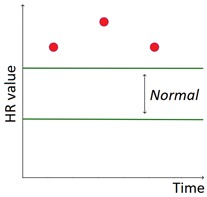
**Complex and/or multivariate**	Rebound Effect (Hypoglycemia followed by Hyperglycemia)	•			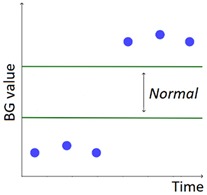
	Dawn Effect (normal BG value at night followed by Hyperglycemia at wake up)	•		•	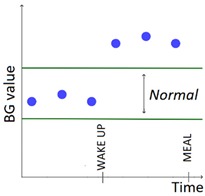
	Tachycardia PRECEDES Hypoglycemia (DURING sleep)	•	•	(•)	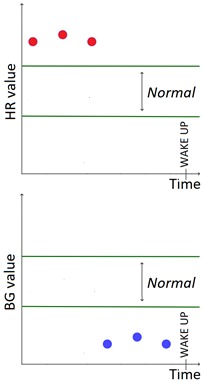
	Hypoglycemia PRECEDES Bradycardia DURING sleep	•	•	•	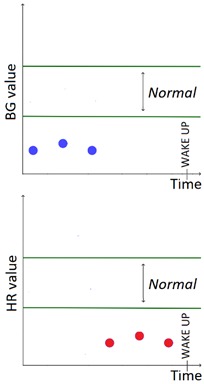

**Table 2:** AID-GM functionalities grouped by type of action. For each functionality, we provide the corresponding users, i.e., patient (P) or clinician (C), and the implementation status compared to the previous version.

Action	Functionality	User		Status
		P	C	
**Set up of the AID-GM account and access**	Access through secure authentication	•	•	
	Request to be enrolled in the clinical center	•		
	View and approval of enrollment request		•	
	Set-up and update of daily habits (i.e., time of meals, wake-up and bedtime for each day of the week)	•		
	Set-up and update of patient-specific thresholds to identify glycemic alterations (i.e., hypoglycemia and hyperglycemia)		•	
	Set-up and update of patient-specific thresholds to identify HR alteration (i.e., tachycardia and bradycardia)		•	New
**Data upload**	Upload of BG monitoring data	•	•	Upgraded
	Consent to download the Fitbit data	•		New
**Data analysis and visualization**	Visualization of BG overall time series, daily trends, and AGP of one patient	•	•	Upgraded
	Visualization of a summary of the most recent hyperglycemic and hypoglycemic episodes	•	•	New
	Visualization of combined BG and HR daily profiles, complemented with information on sleep, workout, meal, and insulin intake	•	•	New
	Visualization of a summary of the physical activity in a selected period	•	•	New
	Visualization of a timeline that shows if the patient is regular in terms of sleep and activity	•	•	New
	Detection and visualization of patterns (Table 1) for one patient	•	•	Upgraded
	Detection and visualization of patterns (Table 1) for a group of patients		•	Upgraded
	Drill-down to the BG and HR profiles related to the time intervals in which a selected pattern occurred	•	•	Upgraded
	Visualization of statistics related to pattern detection for a group of patients		•	
	Visualization of the patients’ list, and list of the recently uploaded data		•	Upgraded
	Visualization of patient’s information (e.g., demographics, contact information, onset date, weight, and thresholds for BG and HR)		•	Upgraded
**Communication between patient and physician**	Request for data visualization	•		
	Notification of data visualization request in the home page		•	

**Table 3:** Characteristics of the sample. To describe the distributions of the subjects’ age and of the duration of the monitoring, we provided the median value for each variable and, in brackets, the interquartile range.

**Sex**	Female: 14 (51.85%), Male: 13 (48.15%)
**Age (years)**	Overall (N = 27):11 [7.5–12.5] Age ≤ 18 (N = 23):9 [7–12] Age > 18 (N = 4):20 [18.75–22]
**Duration of BG monitoring (days) (N = 27)**	97 [65–167]

**Table 4:** Snapshot on the patterns found in the dataset.

Pattern	Total Number of Episodes	Episode Duration in Minutes. Median [Interquartile Range]
BG Decreasing	10,570	75 [45–105]
BG Increasing	10,892	60 [45–91]
Hyperglycemia	8799	165 [60–404]
Severe Hyperglycemia	5842	135 [46–315]
Hypoglycemia	2555	30 [15–60]
Severe Hypoglycemia	516	31 [15–75]
Normal BG	11,631	120 [46–240]

**Table 5:** Number of nights with at least one hypoglycemic episode compared to the total number of nights.

Patient	Number of Nights with Hypoglycemic Episodes	Total Number of Nights
1	13	110
2	7	41
5	12	56
6	9	50
10	10	165
16	3	26

**Table 6:** Number of nighttime episodes of Hypoglycemia and Dawn Effect detected using the profile tag and the Fitbit tag.

Patient	Number of Nighttime Episodes of Hypoglycemia		Number of Episodes of Dawn Effect	
	Fitbit Tag	Profile Tag	Fitbit Tag	Profile Tag
1	16	128	1	7
2	10	17	1	0
5	17	25	2	0
6	10	12	1	0
10	12	18	4	0
16	3	3	1	0

**Table 7:** Mean and standard deviation of the number of actions in the first week compared to all the other weeks.

User	Average Number of Actions in the First Week (SD)	Average Number of Actions in All the Other Weeks (SD)
Physician	67.00 (47.51)	8.61 (7.42)
Patient	21.26 (8.18)	1.17 (0.66)

**Table 8:** Mean and standard deviation of session and training duration.

User	Average Session Duration in Minutes (SD)	Average Training Duration in Minutes (SD)
Physician	9.5 (1.2)	-
Patient	7.3 (3.6)	20.1 (13.5)
